# Editorial: Autoimmune Vasculitis - Advances in Pathogenesis and Therapies

**DOI:** 10.3389/fimmu.2021.720257

**Published:** 2021-06-18

**Authors:** Joshua D. Ooi, Jun Deng, Alexandre W. S. De Souza

**Affiliations:** ^1^ School of Clinical Sciences at Monash Health, Monash University, Clayton, VIC, Australia; ^2^ State Key Laboratory of Oncogenes and Related Genes, Shanghai Cancer Institute, Renji Hospital, School of Medicine, Shanghai Jiao Tong University, Shanghai, China; ^3^ Rheumatology Division, Department of Medicine, Federal University of São Paulo, São Paulo, Brazil

**Keywords:** systemic vasculitis, autoimmune disease, autoimmunity, autoantibodies, T cell targeting, autoepitopes

Systemic vasculitis consists of a collection of heterogenous and rare autoimmune diseases, often with severe life-threatening manifestations. In this Research Topic, we sought to demonstrate the breadth of autoimmune vasculitis, the cutting-edge science being performed to better understand these diseases, and the latest developments in therapies. We were not disappointed. Here, we present 28 articles from across the globe, covering 16 different forms of systemic vasculitis. These include in-depth reviews, novel clinical observations, experimental mouse model studies, as well as the effect of COVID-19 on patients with autoimmune vasculitis. Collectively, the articles in this Topic highlight the complexities of these diseases, their similarities, and the strides that are currently being taken to develop better treatments.

In the ANCA-associated vasculitides (AAV), Li et al. review the genetic associations including both the MHC and non-MHC regions associated with AAV. They demonstrate that the MHC associations provide an additional basis for further dividing the AAV into three subtypes, while specific mutations in the immunoregulatory pathways provide a link to disease pathogenesis. Nozaki provides an up-to-date review on the latest in AAV treatment with biologicals, while O’Sullivan and Holdsworth expound on the relatively new phenomena of NETosis and how understanding its mechanism of injury can lead to the identification of new therapeutic targets. In original AAV research papers, Lin et al. correlate clinical observations of glomerular immune deposition in this traditionally pauci-immune disease with poorer renal survival, and Zeisbrich et al. identify PD-L1 on monocytes as a potential therapeutic target. In the study performed by Zeisbrich et al., the surface expression of the immune checkpoint (PL-L1) on peripheral blood monocytes was assessed in patients with AAV. Monocytes from AAV patients were found to have a lower expression of PD-L1 compared to healthy controls caused by reduced expression of CMTM6, which prevents PD-L1 from degradation. The lower expression of PD-L1 monocytes from AAV patients led to an increased capacity to induce T cells activation and proliferation while inhibiting lysosomal activity increased PD-L1 expression and reduced T cells stimulation by monocytes. This study reveals a potential novel strategy for the treatment of AAV by increasing the expression PD-L1 on monocytes.

In anti-GBM disease, Shen et al. present an impressive study of 60 ultra rare cases of atypical anti-GBM disease, i.e. these patients have linear IgG deposits along their GBM but do not have circulating anti-GBM antibodies in their blood. They found that these atypical anti-GBM patients overall had a less severe renal and pulmonary injury.

In Behcet’s disease, Perazzio et al. present an in-depth and timely review of disease pathogenesis with a focus on the role of innate immunity. The French Behcet’s Network conducted the largest multicenter observational cohort study of apremilast, a phosphodiesterase 4 (PDE4) inhibitor, to treat Behcet’s patients with joint and mucocutaneous symptoms refractory to colchicine and immunosuppressants. Overall, the network found that apremilast was effective in refractory patients.

In Cogan’s syndrome, Venhoff et al. report that Certolizumab pegol, a TNF-α-inhibitor, is effective and well tolerated in two patients with Cogan’s syndrome during pregnancy. This first of its kind study reveals the safety and efficacy of Certolizumab pegol in pregnant patients with inflammatory diseases.

In cutaneous vasculitis, Wang et al. show that HMGB1 blockade in a cutaneous reverse passive Arthus reaction mouse model of disease vastly reduces disease severity. This finding suggests that pursuing an anti-HMGB1 biological treatment in patients would be a promising strategy.

In Giant Cell Arteritis (GCA), we have in this Research Topic three distinct reviews. Akiyama et al. review the latest innate and adaptive immune pathways implicated in GCA; and point to currently available drugs that would be efficacious at inhibiting those pathogenic pathways. Robinette et al. provide a very informative comparison of the similarities and differences in immunopathology between GCA, polymyalgia rheumatica, Takayasu Arteritis and clinically isolated aortitis. Reitsema et al. focus their review on the recent developments in the role of CD8+ T cells in GCA as well as granulomatosis and polyangiitis (GPA).

In IgA nephropathy, Tang et al. apply single-cell RNA sequencing to kidney biopsies from patients to understand the pathways that lead to kidney injury. They show that tubule cells, in particular, were enriched for the TNF signalling, IL-17 signalling and NOD-like receptor signalling inflammatory pathways.

In Kawasaki Disease, Porritt et al. tested the role of anti-IL-1 treatment (anakinra) in suppressing disease using *Lactobacillus casei* cell wall extract (LCWE) injection mouse model. Interestingly, they found that although anakinra administration suppressed *Il6* and *Stat3* gene expression, disease was not attenuated.

Currently, systemic vasculitis is divided into different diseases based on clinical phenotype (1). The PedVas Initiative Investigators performed RNAseq on blood to enable classification of the autoimmune vasculitides based on disease aetiology. This is important because classifying disease based on endotype can inform treatment strategy. Based on whole blood gene expression profiling, the investigators identified two distinct endotypes, neutrophil degranulation and T cell receptor signalling. This promising work can lead the development of targeted therapies that treat the pathogenic mechanisms.

Lastly, in this COVID-19 era, there are two topical papers that look at autoimmune vasculitis and COVID-19. Schramm et al. present a case study of a patient with severe eosinophilic granulomatosis with polyangiitis (EGPA) who contracted COVID-19. They found no major complications despite the patient being highly immunosuppressed. Chen et al. perform a systematic review on cases of COVID-19 associated multisystem inflammatory syndrome in children (MIS-C), which manifests as a Kawasaki disease-like symptoms. Importantly, the authors discuss the immunopathogenesis of COVID-19 associated MIS-C and suggests potential life-saving treatments.

Overall, the papers in this Research Topic highlight the global effort taken to better understand the autoimmune mechanism of systemic vasculitides. As editors, each based in a different continent, we believe that the rarity of these diseases mean that international collaboration is necessary for ultimately developing cures for these severe diseases. Thus, we hope that this Topic serves as an impetus that shows autoimmune vasculitis research is active across the world and encourages vasculitis researchers to initiate collaborations with each other.

## Author Contributions

All authors contributed equally. All authors contributed to the article and approved the submitted version.

## Funding

JDO is funded by an Al and Val Rosenstrauss Fellowship.

## Conflict of Interest

The authors declare that the research was conducted in the absence of any commercial or financial relationships that could be construed as a potential conflict of interest.

